# Conditions and Limits of Calibration-Free Magnetic-Field Measurement: A Minimal Model with In Situ Augmented-Reality Visualization for Wireless Power Transfer

**DOI:** 10.3390/s26154981

**Published:** 2026-08-06

**Authors:** Yunchong Tang, Qiaowei Yuan

**Affiliations:** Faculty of Engineering, Tohoku Institute of Technology, Yagiyamakasumi-cho, Taihaku-ku, Sendai 982-8577, Japan; d232901@st.tohtech.ac.jp

**Keywords:** wireless power transfer, magnetic-field measurement, calibration-free measurement, small-loop magnetic-field probe, augmented reality, electromagnetic compatibility

## Abstract

Accurate magnetic-field characterization is essential for evaluating wireless power transfer (WPT) systems. Conventional near-field measurements require setup-specific probe calibration, increasing experimental complexity. This paper proposes a calibration-free framework based on a minimal small-loop magnetic-field probe model derived from Faraday’s law, where the voltage-to-magnetic-field conversion coefficient is analytically determined from probe geometry and operating frequency. Its validity is examined by comparing an ideal analytical model, a spatially aware Bessel-function-based model, and full-wave simulations. Full-wave simulations indicate that the loop circumference should remain within approximately 0.1λ for the modeled probe, while experimental validation at 13.56 MHz supports the method in the tested WPT configuration. Combined with three-axis measurement and augmented-reality (AR) visualization, the method enables practical three-dimensional (3D) magnetic-field mapping.

## 1. Introduction

Wireless power transfer (WPT) systems have attracted considerable attention for applications such as consumer electronics, electric vehicles, and battery-less sensing systems. In these systems, the spatial distribution of the magnetic field is a key factor that determines power transfer efficiency, electromagnetic compatibility (EMC), and compliance with safety requirements. Therefore, accurate magnetic near-field characterization is essential for WPT system design, optimization, and experimental validation.

Magnetic near-field measurement using small-loop magnetic-field probes is a widely used approach for evaluating field distributions and identifying radiation sources in electronic systems [1,2]. Various magnetic-field probe structures have been developed to improve sensitivity, spatial resolution, bandwidth, and electric-field suppression. For example, ultrawideband printed circuit board (PCB)-based probes have been proposed for broadband near-field measurements [3], and compact shielded-loop probes have been developed to enhance magnetic-field sensitivity while suppressing undesired electric-field coupling [4]. These studies have significantly improved the practicality of magnetic near-field scanning for EMC diagnosis. Recent developments include miniature active and broadband magnetic probes offering enhanced sensitivity and calibrated response [5,6,7], differential and shielded-loop designs aimed at improving unwanted-field rejection and clarifying residual coupling mechanisms [8,9], systematic comparisons of loop configurations in terms of frequency response, spatial resolution, and electric-field suppression [10], and multicomponent differential probes capable of simultaneously sensing orthogonal magnetic-field components [11].

However, accurate quantitative measurement still generally requires probe calibration. In conventional methods, the relationship between the measured probe voltage and the actual magnetic-field strength is obtained using calibration facilities such as transverse electromagnetic (TEM) cells, reference transmission lines, or full-wave-simulation-assisted test structures [12]. Probe calibration and compensation techniques have also been extended to near-field-to-near-field transformation and electromagnetic interference (EMI) source diagnosis [13]. Although these methods are effective, they are often dependent on the specific probe, measurement setup, and calibration environment. As a result, repeated calibration increases the complexity of routine WPT field evaluation.

At the system level, hand-held multi-axis instruments have been developed for in situ assessment of WPT systems and other magnetic near-field sources [14]. Recent studies have further combined three-dimensional magnetic-field modeling with experimental validation [15] and investigated magnetic-field emissions under practical vehicle-mounted WPT operating conditions [16], while smartphone-based AR systems have been used to spatially register and visualize measured magnetic-field data [17]. These developments demonstrate the practical value of vector measurement and spatial visualization, while still relying on calibrated sensing hardware.

To overcome this limitation, this paper proposes a calibration-free magnetic-field measurement framework based on a minimal small-loop magnetic-field probe model derived from Faraday’s law. In the proposed method, the voltage-to-magnetic-field conversion coefficient is analytically determined from the probe geometry and operating frequency. The validity of this coefficient is examined by comparing an ideal analytical model, a spatially aware Bessel-function-based model, and full-wave simulations. Unlike previous studies focused mainly on probe hardware performance or facility-based calibration, this work separates aperture-averaging effects from the full-wave electrical response of the physical loop and integrates the resulting bounded method with multi-axis measurement and in situ AR visualization.

Furthermore, this study clarifies the operational limits of the calibration-free approach. Full-wave simulations indicate that the loop circumference should remain within approximately 0.1λ for the modeled probe to maintain electrically small operation. In addition, three-axis measurement is introduced to reconstruct the total magnetic field under arbitrary polarization. Finally, the proposed method is integrated with an augmented-reality (AR) visualization system to enable intuitive real-time three-dimensional (3D) magnetic-field mapping. Experimental validation at 13.56 MHz demonstrates the feasibility of the proposed framework for practical WPT evaluation and EMC diagnostics without repeated probe calibration.

## 2. Principle of Calibration-Free Measurement

This section describes the theoretical basis of the proposed calibration-free magnetic-field measurement method. According to Faraday’s law, the electromotive force induced in the loop is expressed as

(1) $$V\left(t\right)=-\frac{d\Phi (t)}{dt},$$

where *V* is the induced voltage and Φ denotes the magnetic flux through the loop.

Assuming that the incident magnetic field is perpendicular to the loop surface and spatially uniform over the loop area *S*, the magnetic flux can be expressed as

(2) $$\Phi \left(t\right)=\int_{S}B\left(t\right)\cdot dS=\mu_{0}H\left(t\right)S,$$

where H(t) represents the magnetic field and $\mu_{0}$ is the permeability of free space.

For a time-harmonic field with angular frequency ω, assume $H\left(t\right)=H_{m}\cos\omega t$, where $H_{m}$ is the magnitude of H(t). Substituting (2) into (1) yields

(3) $$V\left(t\right)=\omega \mu_{0}SH_{m}\cos\omega t.$$

Equation (3) indicates that the probe output voltage is directly proportional to the magnetic-field magnitude. Based on this analytical formulation, the voltage-to-field magnitude conversion coefficient $C_{VH}$ of the probe can be obtained as

(4) $$C_{VH,\mathrm{ideal}}=\frac{\left|V(t)\right|}{H_{m}}=\omega \mu_{0}S.$$

Therefore, the magnitude of the magnetic field $H_{m}$ can be estimated directly from the measured probe voltage as

(5) $$H_{m}=\frac{\left|V\right|}{C_{VH,\mathrm{ideal}}}.$$

In the proposed method, $C_{VH}$ is analytically defined from the probe geometry and operating frequency. Therefore, repeated experimental calibration is unnecessary, provided that the incident magnetic field is perpendicular to the loop surface and spatially uniform over the loop area *S*. The next subsection examines the conditions under which these assumptions cease to hold, focusing in particular on the required probe size and on how probe orientation and the polarization of the measured field may affect measurement accuracy.

## 3. Conditions for Calibration-Free Magnetic-Field Measurement

### 3.1. Spatial Uniformity of the Magnetic Field

First, the spatial uniformity of the magnetic field within the probe loop area must be considered. Equation (3) assumes that the magnetic field is constant over the entire loop aperture. In practice, however, in both the near-field and far-field regions of compact sources, the magnetic field may vary significantly over a short spatial distance.

If the magnetic-field distribution varies across the loop surface, the induced voltage should be expressed as

(6) $$V=-\mu_{0}\frac{\partial }{\partial t}\int_{S}H\left(r,t\right)\cdot ds,$$

rather than the simplified expression given in (3), where r represents the position vector. Because the field distribution in the near-field region of the radiation sources is generally complex and strongly dependent on the source geometry, rigorous analytical evaluation becomes difficult. Therefore, here, we focus on the far-field case, where the incident electromagnetic field can be approximated as a plane wave. Based on this condition, the maximum permissible size of the probe loop can be determined to ensure that the uniform-field approximation remains valid.

**Figure 1:**
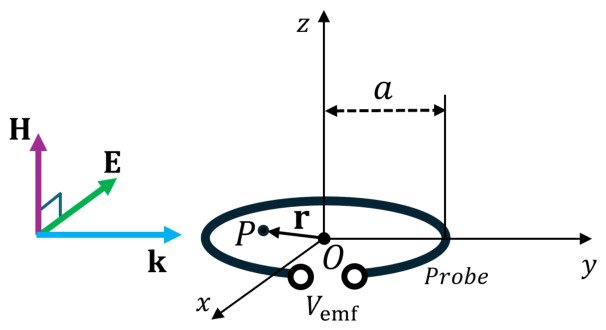
Plane wave propagating along the *y*-axis with a circular loop.

Consider a uniform transverse electromagnetic (TEM) plane wave propagating in free space. The wave propagation is characterized by the wave vector $k=k\hat{y}$. To satisfy the mutually orthogonal TEM condition, the magnetic field vector H and the electric field vector E are linearly polarized along the *z*-axis and *x*-axis, respectively. As shown in Figure 1, a circular magnetic-field probe of radius *a* is placed on the xy-plane centered at the origin. In this configuration, the loop’s geometric normal vector $\hat{n}$ aligns with the incident magnetic field $\left(\hat{n}\|H\right)$, ensuring maximum magnetic flux linkage.

The magnetic field at any spatial point *P*, denoted by the position vector r inside the area enclosed by the loop, can be written as

(7) $$H\left(r,t\right)=H_{m}\cos\left(\omega t-ky\right)\hat{z},$$

where $H_{m}$ denotes the amplitude of the magnetic field vector H.

The total magnetic flux Φ(t) through the loop is found by integrating the incident field H(r,t) over the loop-enclosed area *S*. This integration, which can be analytically simplified using the properties of Bessel functions, yields

(8) $$\Phi \left(t\right)=\int_{S}H\left(r,t\right)\cdot ds=\frac{2\pi \mu_{0}H_{m}}{k}aJ_{1}\left(ka\right)\cos\left(\omega t\right),$$

where k=ω/c is the wavenumber and $J_{1}$ is the Bessel function of the first kind of order one. According to Faraday’s law, the induced electromotive force $V_{\mathrm{bessel}}\left(t\right)$ is the time derivative of this flux:

(9) $$V_{\mathrm{bessel}}\left(t\right)=-\frac{d\Phi (t)}{dt}=2\pi \mu_{0}H_{m}acJ_{1}\left(ka\right)\sin\omega t.$$

The more precise, spatially aware expression for the magnitude conversion factor is denoted as $C_{VH,\mathrm{bessel}}$:

(10) $$C_{VH,\mathrm{bessel}}=\frac{V_{m,\mathrm{bessel}}}{H_{m}}=2\pi \mu_{0}acJ_{1}\left(ka\right).$$

Here, $V_{m,\mathrm{bessel}}=2\pi \mu_{0}H_{m}acJ_{1}\left(ka\right)$ represents the amplitude of $V_{\mathrm{bessel}}\left(t\right)$. When the probe is electrically small (ka≪1), the Bessel function can be approximated by the first term of its Taylor series expansion, $J_{1}\left(ka\right)\approx ka/2$. Substituting this low-frequency approximation into the equation retrieves the ideal conversion factor:

(11) $$C_{VH,\mathrm{bessel}}\approx 2\pi \mu_{0}ac\left(\frac{ka}{2}\right)=\pi a^{2}\mu_{0}c\left(\frac{\omega }{c}\right)=\omega \mu_{0}S=C_{VH,\mathrm{ideal}}.$$

It is important to note that the Bessel-based model asymptotically converges to the simplified ideal model at low frequencies (ka≪1), where the magnetic field remains spatially uniform across the probe area. As frequency increases, the discrepancy between the two analytical models quantifies the aperture-averaging effect under the prescribed plane-wave excitation but does not by itself define the practical upper-frequency limit of the physical loop.

**Figure 2:**
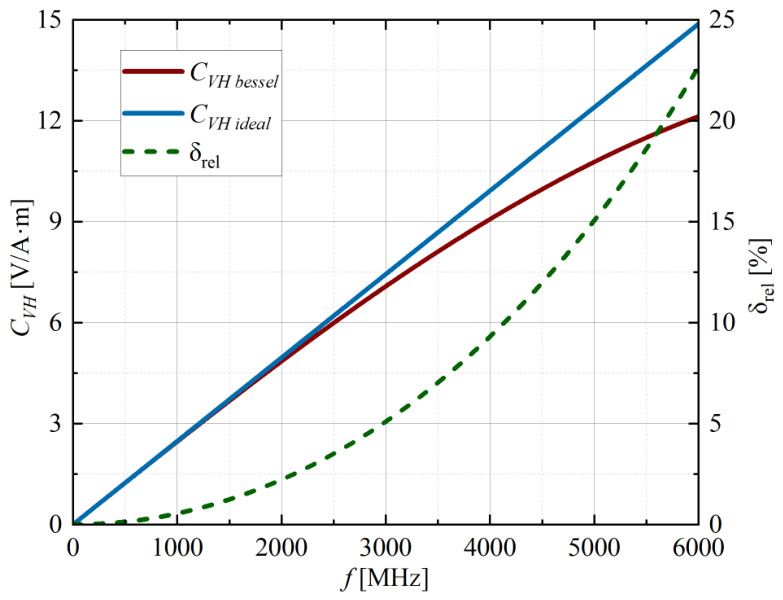
$C_{VH,\mathrm{bessel}}$ and $C_{VH,\mathrm{ideal}}$ versus frequency.

To quantitatively clarify the theoretical limit imposed exclusively by spatial non-uniformity, the ideal conversion factor $C_{VH,\mathrm{ideal}}$ given in (4) is compared with the spatially aware Bessel-function-based model $C_{VH,\mathrm{bessel}}$ derived from (10). As shown in Figure 2, for a probe with a fixed radius of a=10 mm, the deviation between the two conversion factors remains below 5% for frequencies below 3 GHz.

This result confirms that $C_{VH,\mathrm{ideal}}$ under the conventional uniform-field assumption in (4) can be regarded as valid under the spatial condition 2πa/λ<0.63. The plane-wave analysis is used here to isolate the wavelength-dependent aperture-averaging effect and is not intended to provide a universal error bound for nonuniform WPT near fields. Previous finite-area analyses of circular coil probes provide the corresponding treatment for nonuniform magnetic fields [18]. Accordingly, applicability to the present WPT configuration is assessed separately through the geometry-specific full-wave comparison in Section 3.2.

Classical loop-antenna theory imposes a stringent constraint on the electrical size of a loop used as a magnetic-field probe. When the circumference-to-wavelength ratio (2πa/λ) exceeds approximately 0.1, the current distribution can no longer be regarded as uniform because higher-order Fourier current modes are excited [19]. Accordingly, the Faraday-law-based conversion factors, including $C_{VH,\mathrm{ideal}}$ and $C_{VH,\mathrm{bessel}}$, are expected to lose accuracy beyond this electrically small region.

Because the analytical Bessel model neglects the interference of the self-scattered field generated by induced currents, full-wave FEKO simulations are employed to capture these electrodynamic effects and determine the practical electrical-size limit.

**Figure 3:**
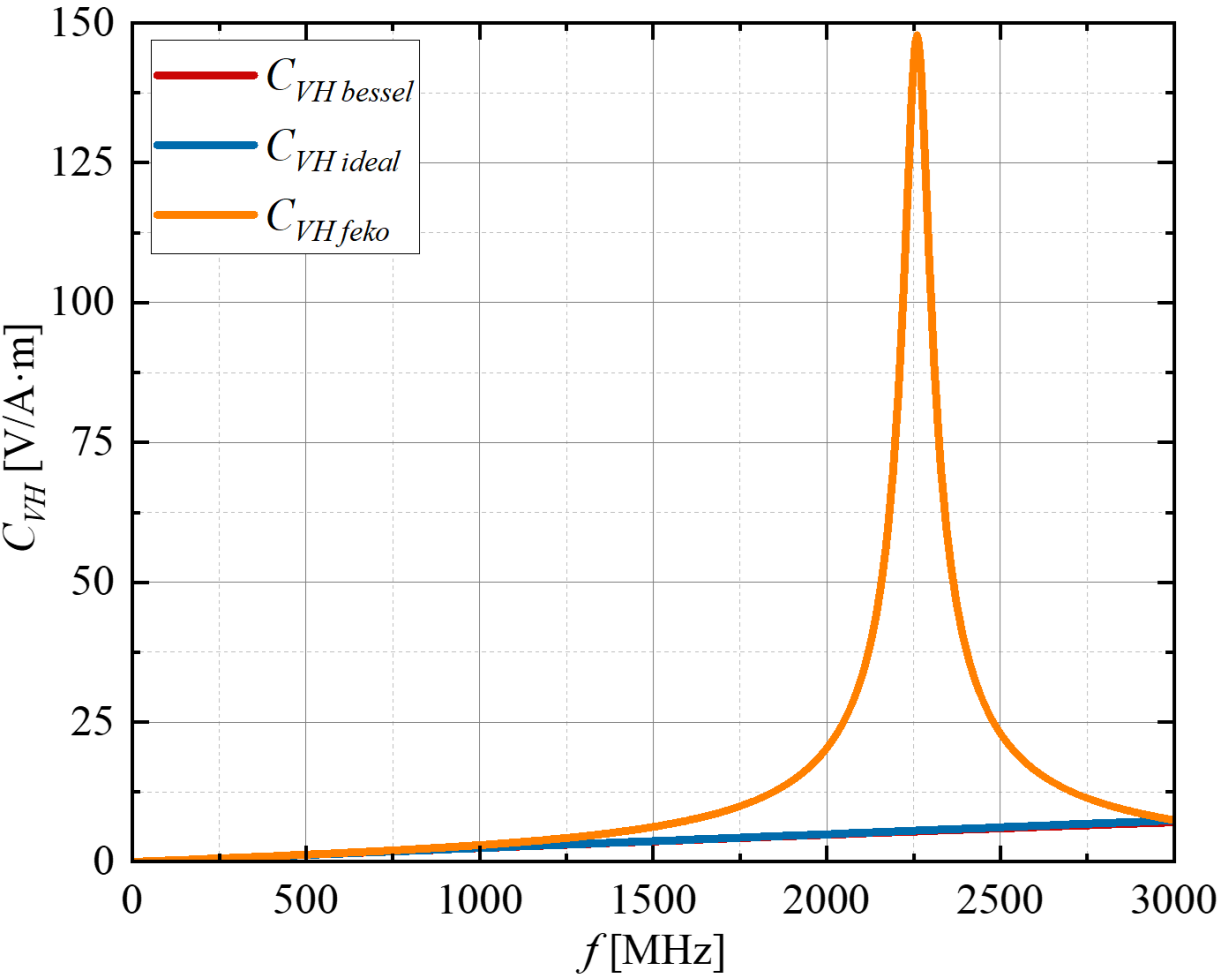
$C_{VH,\mathrm{bessel}}$, $C_{VH,\mathrm{ideal}}$, and full-wave conversion factors versus frequency.

To evaluate this limitation quantitatively, the analytical conversion factors $C_{VH,\mathrm{ideal}}$ and $C_{VH,\mathrm{bessel}}$ are compared with the full-wave numerical factor $C_{VH,\mathrm{FEKO}}$, obtained using the bare-loop model in Figure 1 under identical plane-wave incidence conditions. As shown in Figure 3, all three factors agree well below approximately 530 MHz, with their deviation remaining within 5%. This agreement supports the use of the calibration-free analytical formulation for the modeled loop within this simulated frequency range.

Above this frequency range, however, $C_{VH,\mathrm{FEKO}}$ departs progressively from the analytical predictions, followed by a pronounced resonance around 2.26 GHz. This discrepancy arises because the analytical models consider only the electromotive force induced by the incident magnetic flux, whereas the method-of-moments full-wave solution in FEKO accounts for resonant surface currents and the associated scattered fields. These effects modify the terminal voltage and therefore cannot be represented by a simple Faraday-law-based formulation as also indicated in [20].

In the present simulation, the onset of significant deviation occurs at approximately 530 MHz, corresponding to 2πa/λ≈0.11. This value is consistent with the classical electrically small loop criterion. Therefore, 2πa/λ≤0.11 is adopted as a simulation-supported practical guideline for the modeled physical loop under the analyzed conditions.

### 3.2. Polarization of the Magnetic Field

The polarization of the magnetic field relative to the probe loop area strongly influences the induced voltage. The formulation in the preceding subsection assumes that the dominant magnetic-field component is perpendicular to the probe loop surface.

When the magnetic field forms an angle θ with respect to the normal direction of the loop surface, the effective magnetic flux becomes

(12) $$\Phi =\mu_{0}SH_{m}\cos\theta ,$$

and the induced voltage is reduced accordingly. As a result, probe misalignment leads to a systematic underestimation of the magnetic-field magnitude.

Therefore, the allowable angular deviation between the probe orientation and the magnetic-field direction must be evaluated to maintain sufficient measurement accuracy.

**Figure 4:**
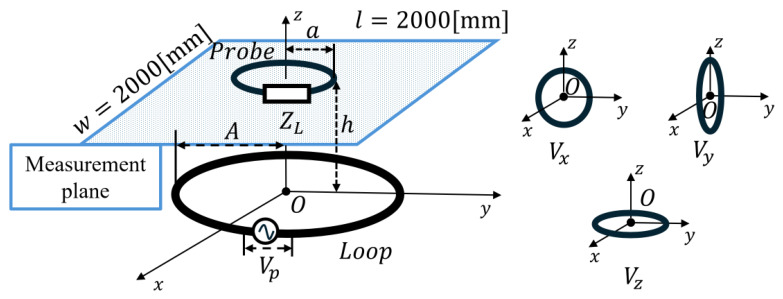
Schematic of the three-axis magnetic-field measurement model.

To quantify the measurement errors caused by highly divergent and arbitrarily polarized fields, which are typical in practical WPT applications, a full-wave electromagnetic simulation model as shown in Figure 4 was developed. A large single-turn coil with a radius of A=352 mm was employed as the radiation source because it naturally generates a magnetic field with strong multidirectional components, especially near its outer periphery. The radiating coil, excited at 13.56 MHz, was placed on the xy-plane. The radiated magnetic field over a 2000×2000 mm area at z=350 mm was evaluated using an electrically small magnetic-field probe with a radius of a=10 mm and a spatial resolution of 100 mm. The detailed simulation model parameters are summarized in Table 1.

The excitation frequency was set to 13.56 MHz, for which the electrical size of the probe was $2\pi a/\lambda \approx 2.84\times 10^{-3}\ll 0.11$. This confirms electrically small-loop operation, while the applicability of the ideal conversion factor to the present nonuniform WPT field is evaluated separately through the geometry-specific full-wave comparison presented below. Accordingly, $C_{VH,\mathrm{ideal}}\approx 0.03344\;\left[V\cdot m/A\right]$ in (4) is used to convert the calculated induced voltage into the reconstructed magnetic-field strength. To accurately obtain the induced probe voltage, the load impedance at the receiving port, $Z_{L}$, was set to 100 MΩ. Since the magnetic field has three components, the induced voltages $V_{x}$, $V_{y}$, and $V_{z}$ were calculated for the probe in three orthogonal orientations to reconstruct $H_{x}$, $H_{y}$, and $H_{z}$.

**Figure 5:**
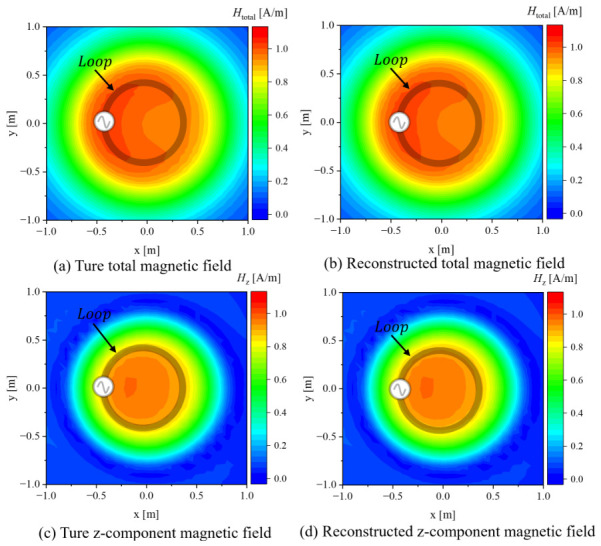
Reference and analytically reconstructed magnetic-field distributions.

Figure 5 compares the true and analytically reconstructed magnetic-field distributions. The total magnetic field is defined as $|H_{\mathrm{total}}|=\sqrt{H_{x}^{2}+H_{y}^{2}+H_{z}^{2}}$, which is obtained by vectorially combining the three orthogonal components. As shown in Figure 5a,b, the reconstructed total magnetic field agrees well with the true total magnetic field over the entire observation region. In contrast, Figure 5c,d show only the *z*-component of the magnetic field, $H_{z}$, and the reconstructed $H_{z}$ distribution in Figure 5d agrees well with the analytically calculated $H_{z}$ distribution in Figure 5c. However, $H_{z}$ alone represents the total magnetic field accurately only in the central region above the coil, where the field is predominantly *z*-polarized. In the outer region of the coil, the transverse components, $H_{x}$ and $H_{y}$, become significant, indicating that three-axis measurement is necessary for accurate near-field characterization.

### 3.3. Summary of the Analyzed Operational Boundaries

The preceding analytical and numerical results identify two operational conditions for calibration-free magnetic-field measurement:

- Electrical-size guideline: The electrical size of the probe must satisfy 2πa/λ≤0.11. This condition is derived from the requirement of maintaining a nearly uniform current distribution along the loop, rather than from an empirical fitting criterion.
- Polarization limit: A multi-axis measurement approach is required to accurately reconstruct the total magnetic field, $|H_{\mathrm{total}}|=\sqrt{H_{x}^{2}+H_{y}^{2}+H_{z}^{2}}$, without polarization mismatch.

Under these electrical-size and directional conditions, the calibration-free methodology can be evaluated as an engineering tool for quantitative near-field characterization. The subsequent experiment tests the method at 13.56 MHz; it does not experimentally verify the complete simulation-derived frequency boundary.

## 4. System Integration: Combining the Calibration-Free Probe with Augmented-Reality Magnetic-Field Measurement

### 4.1. System Architecture and Spatial Tracking

**Figure 6:**
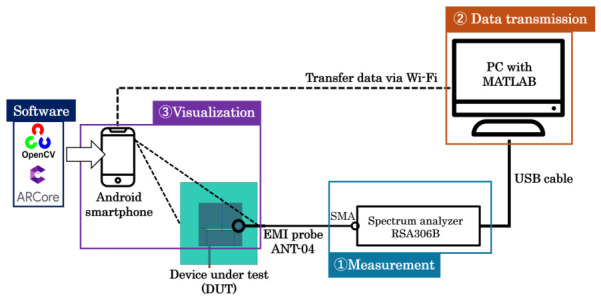
Architecture of the AR-based magnetic-field measurement system.

To address the spatial and directional limits discussed in Section 3, the proposed calibration-free compact probe was integrated with an augmented-reality (AR) visualization system. As shown in Figure 6, the system uses an electrically small magnetic-field probe connected to a spectrum analyzer as the measurement front end, together with a data processing backend and an AR-enabled smartphone frontend.

This system leverages OpenCV-based computer vision algorithms to track a planar ArUco marker attached to the probe. In our laboratory setup, the average spatial localization error was less than 1 cm at a camera distance of 70 cm. By continuously aligning the probe’s coordinate frame with the real-world environment, the magnetic field strength calculated using $C_{VH,\mathrm{bessel}}$ is dynamically mapped and rendered on the smartphone screen via AR technology.

**Figure 7:**
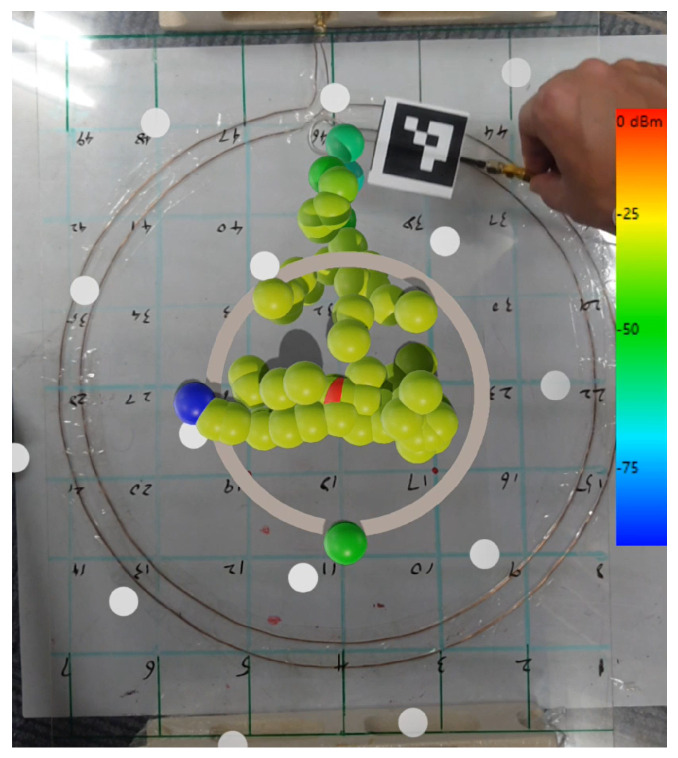
Demonstration of AR-based magnetic-field measurement.

Figure 7 displays the real-time AR visualization of the *z*-component magnetic field distribution seamlessly overlaid onto the physical WPT coil. The colored spheres show the magnetic field intensity in the camera view, where blue indicates low intensity and red indicates high intensity. This intuitive spatial mapping substantially reduces the complexity of conventional EMC scanning procedures.

### 4.2. AR-Based WPT Magnetic-Field Measurement

To evaluate the calibration-free methodology within the analyzed conditions, an experimental frequency of 13.56 MHz, which is widely used in WPT systems, was selected.

**Figure 8:**
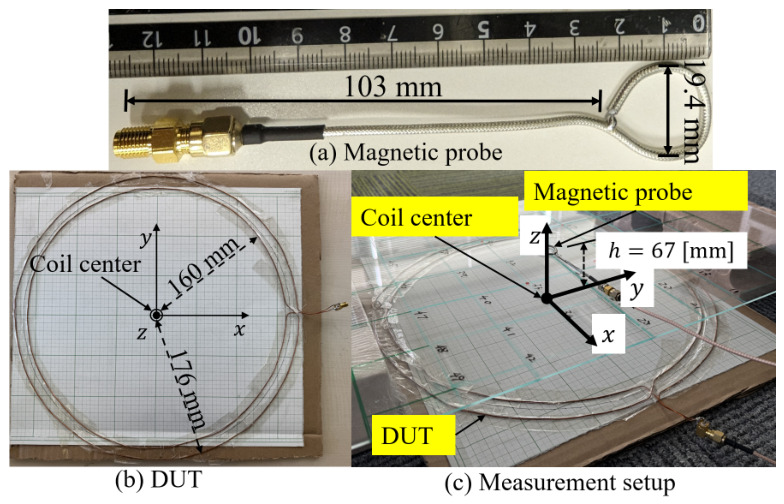
Fabricated magnetic-field probe, device under test (DUT), and measurement setup.

Magnetic-field measurements were then performed on a custom WPT system comprising a two-turn transmitting coil as the device under test (DUT) as shown in Figure 8b. For the magnetic-field pickup, a shielded magnetic-field probe was fabricated using a semi-rigid coaxial cable as shown in Figure 8a [21]. The probe radius is a=10 mm, which places the probe well below the simulation-supported guideline 2πa/λ≤0.11. Furthermore, a cut-out was introduced in the probe shield at the neck of the loop. While this coaxial shielding structure is intended to reduce parasitic electric-field coupling in the tested near-field WPT setup, the asymmetric cut-out placement results in an inherently unbalanced configuration. Consequently, residual electric-field coupling and common-mode pickup caused by this asymmetry cannot be completely excluded. The development of a strictly balanced shielded loop (e.g., with an apex cut-out) or an optically isolated sensor to further suppress these parasitic effects remains a subject for future work.

**Figure 9:**
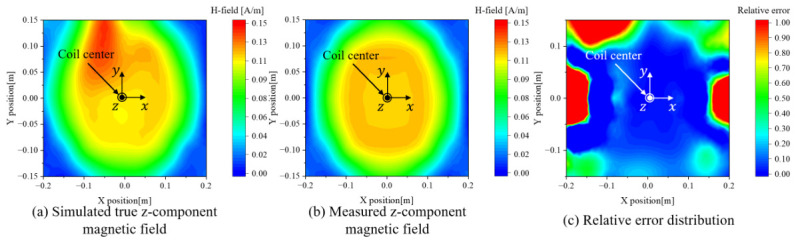
Comparison of simulated and measured *z*-component magnetic-field distributions at z=67 mm.

As illustrated in Figure 8c, the radiating two-turn loop is placed on the xy-plane as the radiating source, while the 400×300 mm^2^ measurement plane is set at z=67 mm. To quantitatively evaluate the proposed method, the AR-mapped field data were extracted and compared with the full-wave simulation results along a specified axis above the coil, as plotted in Figure 9. The validation dataset used for this comparison originates from our preliminary ISAP 2024 conference study [22]; Figure 9 was newly generated from the source data for the present analysis using the corrected theoretical conversion coefficient. Close agreement is observed between the measured and simulated distributions of the *z*-component magnetic field, particularly within the primary region of interest directly above the coil. Quantitative analysis shows that the relative error remains predominantly below 10% within a circular region with a radius of 0.1 m from the coil center.

Although higher relative errors appear at the outer extremities beyond a circular region with a radius of 0.1 m, this is a common artifact in practical measurements. In these peripheral regions, the absolute magnetic-field intensity decreases; therefore, ambient environmental noise and the limited dynamic range of the measurement instrument disproportionately increase the calculated relative error. These results support the use of the proposed analytical minimal model for quantitative near-field characterization in the tested 13.56 MHz configuration without repeated frequency-dependent facility calibration.

## 5. Conclusions

This paper proposed and evaluated a calibration-free magnetic-field measurement framework integrated with an augmented-reality (AR) visualization system. The minimal analytical model derived from Faraday’s law determines the voltage-to-field conversion from probe geometry and frequency and can avoid repeated facility-based calibration when the stated electrical-size, field-distribution, and orientation assumptions are satisfied.

The Bessel-function-based analysis isolates aperture-averaging effects, whereas the FEKO full-wave simulation accounts for the electrical response of the physical loop. For the modeled a=10 mm loop, the full-wave results indicate the onset of significant deviation at approximately 530 MHz, corresponding to 2πa/λ≈0.11. This value is treated as a simulation-supported guideline under the analyzed conditions rather than as an experimentally verified frequency cutoff.

Experimental validation at 13.56 MHz showed close agreement between the measured and simulated magnetic-field distributions within the effective measurement region of the tested WPT system. Integration with AR technology enabled intuitive real-time three-dimensional mapping. These results support the feasibility of the framework for WPT evaluation under the reported conditions, while multi-frequency experimental verification of the simulation-derived boundary remains a subject for future work.

## Figures and Tables

**Table 1:** Parameters of the radiation loop and probe.

Parameter	Value
Probe radius, *a* [mm]	10
Loop radius, *A* [mm]	352
Probe height, *h* [mm]	350
Port impedance, $Z_{L}$ [MΩ]	100
Voltage source magnitude, $V_{p}$ [V]	1000
Voltage source frequency, *f* [MHz]	13.56

## Data Availability

The original contributions presented in this study are included in the article. Further inquiries can be directed to the corresponding author.
